# Coronaviruses in wild rodent and eulipotyphlan small mammals: a review of diversity, ecological implications and surveillance considerations

**DOI:** 10.1099/jgv.0.002130

**Published:** 2025-07-11

**Authors:** Simon P. Jeeves, Jonathon D. Kotwa, David L. Pearl, Bradley S. Pickering, Jeff Bowman, Samira Mubareka, Claire M. Jardine

**Affiliations:** 1 Department of Pathobiology, University of Guelph, Guelph, Ontario, Canada; 2 Sunnybrook Research Institute, Toronto, Ontario, Canada; 3 Department of Population Medicine, University of Guelph, Guelph, Ontario, Canada; 4 National Centre for Foreign Animal Disease, Canadian Food Inspection Agency, Winnipeg, Manitoba, Canada; 5 Department of Medical Microbiology and Infectious Diseases, University of Manitoba, Winnipeg, Manitoba, Canada; 6 Wildlife Research and Monitoring Section, Ontario Ministry of Natural Resources, Peterborough, Ontario, Canada; 7 Environmental and Life Sciences Graduate Program, Trent University, Peterborough, Ontario, Canada; 8 Department of Laboratory Medicine and Pathobiology, University of Toronto, Toronto, Ontario, Canada; 9 Canadian Wildlife Health Cooperative, Ontario-Nunavut, Department of Pathobiology, University of Guelph, Guelph, Ontario, Canada

**Keywords:** coronaviruses, Eulipotyphla, rodent, small mammals, surveillance, wildlife

## Abstract

Coronaviruses are abundant and diverse RNA viruses with broad vertebrate host ranges. These viruses include agents of human seasonal respiratory illness, such as human coronaviruses OC43 and HKU1; important pathogens of livestock and domestic animals such as swine acute diarrhoea syndrome coronavirus and feline coronavirus; and human pathogens of epidemic potential such as SARS-CoV, MERS-CoV and SARS-CoV-2. Most coronavirus surveillance has been conducted in bat species. However, small terrestrial mammals such as rodents and eulipotyphlans are important hosts of coronaviruses as well. Although fewer studies of rodent and eulipotyphlan coronaviruses exist compared to those of bats, notable diversity of coronaviruses has been reported in the former. No literature synthesis for this area of research has been completed despite (a) growing evidence for a small mammal origin of certain human coronaviruses and (b) global abundance of small mammal species. In this review, we present an overview of the current state of coronavirus research in wild terrestrial small mammals. We conducted a literature search for studies that investigated coronaviruses infecting rodent and eulipotyphlan hosts, which returned 63 studies published up to and including 2024. We describe trends in coronavirus diversity and surveillance for these studies. To further the examination of the interrelatedness of these viruses, we conducted a phylogenetic analysis of coronavirus whole genomes recovered from rodent and eulipotyphlan hosts. We discuss important facets of terrestrial small mammal coronaviruses, including evolutionary aspects and zoonotic spillover risk. Lastly, we present important recommendations and considerations for further surveillance and viral characterization efforts in this field.

## Introduction

Infectious disease is a major cause of human morbidity and mortality worldwide. Many emerging and re-emerging infectious diseases are zoonotic: transmitted from animal hosts to a human host. Zoonotic pathogens are now thought to cause at least two out of every three novel human diseases and are comprised of a diverse range of infectious agents, including viruses, bacteria, fungi, helminths, protozoa and prions [1,2]. The emergence of these pathogens is expected to continue or even increase as climate change, globalization and urbanization [3] redefine existing disease transmission pathways and create opportunities for the spillover of zoonotic pathogens between animals and humans [3,4].

### Wildlife and zoonotic disease

The majority of emerging zoonoses have had their origins traced back to animal sources, primarily wildlife [1,2]. This is particularly the case for zoonoses with epidemic or pandemic potential, such as plague (*Yersinia pestis*), henipaviruses, ebolaviruses, influenza viruses and, most notably, SARS-CoV and SARS-CoV-2 [2]. Although vertebrate animals of all classes carry agents of disease that may be transmitted to humans under the right circumstances, there is a greater abundance of pathogens with spillover potential in mammalian and avian species [5,7].

Bats (order Chiroptera) and non-human primates harbour the highest density of known zoonotic pathogens, but smaller terrestrial mammals such as rodents, shrews and hedgehogs carry the greatest diversity of known zoonoses and have a high proportion of species known to act as hosts for such pathogens [5,9].

### Wild terrestrial small mammals and zoonotic disease

Mammals in the order Rodentia – the most diverse, numerous and widely disseminated of mammalian orders – play host to many agents of emerging zoonoses [8,10]. Rodents are found on all continents but Antarctica [11,13] and inhabit a wide range of habitat types, from forests and grasslands to city alleyways and urban green spaces [12]. These factors, in combination with a rapid generation time, make rodents a leading priority for the ecological study of a wide range of zoonotic agents [8].

It has been estimated that nearly 11% of rodent species are hosts of zoonotic pathogens – the highest proportion out of all mammalian orders [8]. Although rats and other murids have long been associated with zoonotic diseases, including plague, scrub typhus and other historically important diseases, advances in diagnostic methods have allowed for discoveries of novel viruses, bacteria and parasites in rodent hosts on an unprecedented scale. Rodent species have already been implicated as reservoirs for many important emerging zoonotic agents, including species of *Leptospira* bacteria, hantaviruses, *Francisella tularensis*, *Mammarenavirus choriomeningitidis*, *Mammarenavirus lassaense* and *Streptobacillus moniliformis*, the etiological bacterium of rat bite fever [10].

Eulipotyphla, another order of small mammals, includes animals that are typically small in body mass, including hedgehogs and gymnures (family Erinaceidae), moles (family Talpidae), solenodons (family Solenodontidae) and shrews (family Soricidae) [14]. Eulipotyphlans have comparable species diversity to bats and primates but, interestingly, contain few known zoonotic hosts [8,14]; however, there is a paucity of studies involving eulipotyphlan wildlife [8]. Recent identification of shrews as hosts for *Parahenipavirus langyaense*, a novel orthoparamyxovirus closely related to other henipaviruses of major zoonotic concern, warrants further study of this group [15]. Studies of hedgehogs have focused on zoonotic risk from pets, but more recently, a review has described an assortment of zoonotic agents carried by wild hedgehogs as well [16,17].

Here, we define small mammals as terrestrial species within orders Rodentia and Eulipotyphla with an average body mass of up to 5 kg [12,18]. Notwithstanding their phylogenetic differences, the two described mammalian taxa have similar global distributions and share important ecological roles, such as seed dispersal, soil aeration, nutrient cycling and serving as prey species for multiple predators [11,13, 19,21]. Rodents are primarily herbivores/granivores, whereas shrews are primarily insectivores, but both taxa often exhibit omnivorous foraging behaviours [22]. The majority of rodents and eulipotyphlans are highly terrestrial, living in burrows or nests on the ground [11,13]. These animals have relatively fast generation times, allowing for regular introduction of immunologically naïve hosts to their local populations [12,13]. Rodents and eulipotyphlans are diverse in their species and the habitats they occupy; both often live directly within urban and periurban environments as well as in naturalized areas and rural habitats [12]. Naturalized areas such as forests and fields are typically inhabited by species of terrestrial small mammals that are native to the region in question. In agricultural contexts, an abundance of grain and related food sources can attract populations of both native and non-native small mammals [12,22]. In urban and periurban environments worldwide, high proportions of built-up area and heavy landscape fragmentation provide an attractive habitat for urban exploiter rodents such as Norway rats (*Rattus norvegicus*) and house mice (*Mus musculus*) [23]. Islands of naturalized areas such as parks and green spaces in cities can allow habitation by native species as well [23]. In any case, the remarkable adaptability of rodents and eulipotyphlans allows many species to inhabit a wide variety of environmental contexts.

Overall, this means that wild terrestrial small mammals often cohabitate with a variety of other species, including humans, domestic animals and livestock. Taken together, species from the orders Rodentia and Eulipotyphla exhibit two qualities important to pathogen ecology: (1) overlapping species distributions that enable potential transmission of a variety of pathogens between animal hosts and environments and (2) the ability to thrive in human-modified environments, increasing potential risk for zoonotic transmission.

### Coronaviruses

Coronaviruses (CoVs) are causative agents of primarily respiratory and gastrointestinal infections in humans and a wide range of vertebrate animals [24]. Coronaviruses are of utmost importance as emerging pathogens of epidemic and pandemic potential, as has been demonstrated by SARS-CoV, MERS-CoV and SARS-CoV-2. All three of these viruses, in addition to seasonal human CoVs, have contributed to significant human and animal morbidity, mortality and economic burden on a global scale [25,27].

Coronaviruses possess monopartite, single-stranded positive-sense RNA genomes with sizes of ~27–32 kb [24]. Coronavirus genomes are the largest and most complex of all known RNA viruses. They are prone to recombination events and exhibit high mutation rates, although genome replication infidelity is moderated by exoribonuclease proofreading activity which is believed to support the stability and viability of their large RNA genomes [24,28]. The swift evolutionary capability of RNA viruses is a critical facet of their repeated emergence as novel human pathogens. Coronaviruses belong to the family *Coronaviridae* within the order *Nidovirales* [24]. The family *Coronaviridae* is composed of three subfamilies: *Orthocoronavirinae*, which contains most species, *Letovirinae* and *Pitovirinae* [24,29, 30]. The subfamily *Orthocoronavirinae* is currently organized into four genera: *Alphacoronavirus* (alpha-CoVs), *Betacoronavirus* (beta-CoVs), *Deltacoronavirus* (delta-CoVs) and *Gammacoronavirus* (gamma-CoVs) [24]. Although CoVs have been recovered from a diverse variety of vertebrate taxa, the majority infect mammals and birds. Based on molecular clock dating analysis of CoV genomes, researchers have suggested that alpha-CoVs and beta-CoVs are associated with mammalian coevolution, while those of *Gammacoronavirus* and *Deltacoronavirus* are primarily avian in origin [24,31, 32].

Human CoVs are all believed to have originated in non-human animal hosts [33,35]. All human CoVs are either alpha-CoVs or beta-CoVs [24]. As alpha- and beta-CoVs are associated with mammalian hosts [31,32], there has been a focus in the literature on examining wild mammals and the CoVs they may carry [24]. Alphacoronaviruses are organized into 15 subgenera, including *Duvinacovirus* and *Setracovirus* which contain human CoVs 229E and NL63, respectively [24]. Betacoronaviruses are organized into five subgenera: *Embecovirus*, *Hibecovirus*, *Merbecovirus*, *Nobecovirus* and *Sarbecovirus* [24]. *Betacoronavirus pandemicum* [previously *Severe acute respiratory syndrome-related coronavirus* (SARS-CoV)], *Betacoronavirus cameli* [previously *Middle East respiratory syndrome-related coronavirus* (MERS-CoV)] and, most recently, severe acute respiratory syndrome coronavirus 2 (SARS-CoV-2) – the cause of Coronavirus disease 2019 (COVID-19) – are examples of CoVs responsible for significant human epidemics [24,36]. The emergence of SARS-CoV, MERS-CoV and SARS-CoV-2 all within the last two decades is a testament to the ubiquitous and rapidly evolving nature of CoVs as human pathogens. Studies have suggested that SARS-CoV, MERS-CoV and SARS-CoV-2 all possess bat CoV ancestry [37,39]. Much attention has consequently been given to CoVs circulating in bats, but the origins of other clinically relevant CoVs have either been linked to different mammalian taxa or remain unclear [34]. Other mammals identified as likely reservoirs or intermediate hosts involved in human spillover or spillback of CoVs have included viverrids, mustelids, cervids, caniforms, cattle, swine and rodents [34,40,42]. Additionally, surveillance studies in *Erinaceus* hedgehog species have found viruses with high similarity to MERS-CoV – a virus that is typically found in camelids [43,45].

Small mammal species play a role in the ecology and evolution of CoVs with implications for both human and animal health. Yet, to the best of our knowledge, no synthesis of this research area exists. In this review, we aim to describe the body of existing research that has begun to illustrate the diversity of CoVs naturally found in terrestrial small mammal hosts from around the world. Furthermore, we discuss important and ecologically relevant aspects of specific CoVs recovered from rodents and eulipotyphlans. Lastly, we summarize trends in existing studies and present an argument for the importance of robust, comprehensive and continuous surveillance of CoV surveillance in terrestrial small mammal species.

## Methods

### Literature search

We used the following search string to identify existing studies that aimed to identify undescribed CoVs in wild terrestrial small mammals: (small mammal OR rodent* OR shrew OR hedgehog OR Eulipotyphla*) AND (coronavirus OR *Coronaviridae*) AND wild*. This search string was used in PubMed, Web of Science and the University of Guelph library’s Omni search tool (https://www.lib.uoguelph.ca/). To further expand the scope of our search, we used the database of zoonotic and vector-borne viruses (ZOVER) (https://www.mgc.ac.cn/cgi-bin/ZOVER/main.cgi) [46]. ZOVER is a manually compiled, regularly updated database of virus sequences found in NCBI GenBank that were recovered from rodent, bat, tick or mosquito hosts [46]. All available references in the rodent database under ‘*Coronaviridae*’ were included; note that 949/1626 GenBank records displayed in ZOVER were listed as ‘unpublished’. Results of the initial search string were filtered to only include those from peer-reviewed journals and then manually screened for studies that utilized a pan-*Coronaviridae* approach – typically PCR or metagenomics-based – for testing biological samples obtained from wild terrestrial small mammals. Studies published in the years leading up to and including 2024 were considered; studies not available in English were excluded. The retained articles were then subject to citation searching to identify additional studies. After removal of duplicates, 63 studies were included here (Table 1). Note that this review is not intended to be a comprehensive scoping review of the literature. While some metagenomics-based studies were captured in our search, it is likely inexhaustive – studies that use a metagenomics approach for identifying novel viruses would not have been captured by our search string if no *Coronaviridae* or *Coronaviridae*-like sequences were reported.

**Table 1.** Previous studies into CoV diversity in wild rodent and eulipotyphlan small mammals up to and including 2024 (*n*=63)

Authors	Year of publication	Continent	Country	Animal collection method^1^	Sample type^2^	Small mammal order tested^3^	Rodent families tested	Eulipotyphlan families tested	CoV+animals (%)^4^	CoV genus detected^4^
Phan *et al*. [Bibr R63][63]^11^	2011	North America	USA	T(L)	F	Rodentia	Cricetidae		n/a ^12^	nd ^5^
Corman *et al*. [Bibr R43][43]	2014	Europe	Germany	V	F, T	Eulipotyphla		Erinaceidae	146/248 (58.9)	Beta
Wang *et al*. [Bibr R64][64]	2015	Asia	China	T(L)	F, T	Rodentia	Cricetidae, Muridae		30/1,465 (2.0)	Alpha, Beta
Lau *et al*. [Bibr R65][65]	2015	Asia	China	nr	T	Rodentia	Muridae		3/659 (0.5)	Beta
						Eulipotyphla		Soricidae	0/5 (0.0)	nd
Tsoleridis *et al.* [Bibr R129][129]	2016	Europe	UK, Poland	T(U), V	T	Rodentia	Cricetidae, Muridae		10/810 (1.2)	Alpha
						Eulipotyphla		Soricidae	1/3 (33.3)	Alpha
Ge *et al*. [Bibr R114][114]	2017	Asia	China	V	T	Rodentia	Cricetidae, Muridae		23/177 (13.0)	Alpha, Beta
Anthony *et al*. [Bibr R125][126]	2017	Africa, Asia, North America, South America	Bolivia, Brazil, Cambodia, Cameroon, China, DRC^6^, Gabon, Indonesia, Laos, Malaysia, Mexico, Nepal, Peru, Mexico, ROC, Rwanda, Tanzania, Thailand, Uganda, Vietnam	T(L)	S, T, O	Rodentia	Not reported		11/3,387 (0.3)^7^	Beta^7^
						Eulipotyphla		Not reported		
Monchatre-Leroy *et al*. [Bibr R130][130]	2017	Europe	France	E, T(U), V	F, T	Rodentia	Cricetidae, Muridae		21/330 (6.4)	Beta
						Eulipotyphla		Erinaceidae	37/74 (50.0)	Beta
Wang *et al.* [Bibr R75][75]	2017	Asia	China	T(L)	T	Eulipotyphla		Soricidae	24/266 (9.0)	Alpha
Guan *et al*. [Bibr R131][131]	2017	Asia	China	T(U)	F	Eulipotyphla		Soricidae	2/40 (5.0)	Alpha
Williams *et al.* [Bibr R132][132]^13^	2018	North America	USA	T(L)	F	Rodentia	Muridae		n/a ^12^	Beta
Berto *et al*. [Bibr R121][122]	2018	Asia	Vietnam	MF	F	Rodentia	Muridae		12/270 (4.4)	Beta
Wu, Lu, Du *et al*. [Bibr R76][76]^11^	2018	Asia	China	T(L)	S	Rodentia	Cricetidae, Dipodidae, Muridae, Sciuridae		118/2752 (4.3)	Alpha, Beta
						Eulipotyphla		Soricidae	5/224 (2.2)	Alpha
Phan *et al*. [Bibr R122][123]^11^	2018	Asia	Vietnam	T(U), MF	F	Rodentia	Muridae		11/391 (2.8)	Beta
Saldanha *et al.* [Bibr R66][66]^13^	2018	Europe	UK	V	F, T	Eulipotyphla		Erinaceidae	38/351 (10.8)^8^	Beta
Lau *et al*. [Bibr R44][44]	2019	Asia	China	nr	T	Rodentia	Muridae		0/151 (0.0)	nd
						Eulipotyphla		Erinaceidae, Soricidae	2/56 (3.6)	Beta
Onyuok, Hu *et al*. [Bibr R133][133]	2019	Africa	Kenya	T(L)	F, T, O	Rodentia	Gliridae, Muridae, Nesomyidae, Spalacidae		0/55 (0.0)	nd
						Eulipotyphla		Erinaceidae	0/1 (0.0)	nd
Delogu *et al.* [Bibr R67][67]^13^	2020	Europe	Italy	V	F	Eulipotyphla		Erinaceidae	14/24 (58.3)	Beta
Huong *et al*. [Bibr R98][98]^13^	2020	Asia	Vietnam	E, MF	F, S, T, O	Rodentia	Muridae, Spalacidae, Sciuridae		266/1,131 (23.5)	Alpha, Beta
McIver *et al*. [Bibr R123][124]	2020	Asia	Laos	P, T(LK), V	B, F, S, T, O	Rodentia	Dyatomyidae, Muridae, Sciuridae		12/851 (1.4)	Beta
Wang *et al.* [Bibr R84][84]	2020	Asia	China	T(L)	T	Rodentia	Cricetidae, Muridae		39/696 (5.6)	Alpha, Beta
Wolking *et al.* [Bibr R134][134]	2020	Asia	Nepal	T(L)	F, S	Rodentia	Muridae		2/98 (2.0)	Beta
						Eulipotyphla		Soricidae	0/313 (0.0)	nd
Vandegrift *et al.* [Bibr R135][135]^11^	2020	North America	USA	T(U)	B	Rodentia	Cricetidae		n/a^12^	nd ^5^
Zhu *et al.* [Bibr R100][100]^11^	2021	Asia	China	T(L)	F	Rodentia	Cricetidae, Muridae		4/79 (5.1)^9^	Beta, Delta
Monastiri *et al.* [Bibr R136][136]	2021	Africa	Canary Islands	T(L)	F	Rodentia	Muridae		11/260 (4.2)	Beta
Li *et al.* [Bibr R45][45]	2021	Asia	China	T(L)	T	Eulipotyphla		Erinaceidae	5/51 (9.8)	Beta
Li *et al*. [Bibr R99][99]^13^	2021	Asia	China	T(L)	S, T	Rodentia	Muridae, Spalacidae		35/294 (11.9)	Alpha, Beta
						Eulipotyphla		Soricidae	0/3 (0.0)	nd
Kumakamba *et al.* [Bibr R124][125]	2021	Africa	DRC, ROC^8^	MF, T(L), V	S, T	Rodentia	Bathyergidae, Muridae, Nesomyidae, Sciuridae, Thryonomyidae		2/1,347 (0.1)	Alpha
						Eulipotyphla		Soricidae	0/22 (0.0)	nd
Ip *et al.* [Bibr R106][106]	2021	North America	USA	T(L)	T	Rodentia	Cricetidae, Muridae		16/98 (16.3); nine indeterminate	Alpha, Beta
Wu, Han, Liu *et al*. [Bibr R137][137]^11^	2021	Asia	Cambodia, Laos, Thailand	T(U)	T	Rodentia	Muridae		9/3,191 (0.3)	Beta
						Eulipotyphla		Soricidae	0/93 (0.0)	nd
Tirera *et al.* [Bibr R138][138]^11, 13^	2021	South America	French Guiana	T(L)	B, T	Rodentia	Cricetidae, Echimyidae		n/a ^12^	nd
Ntumvi *et al.* [Bibr R139][139]	2022	Africa	Cameroon	T(L), V	S, T	Rodentia	Muridae, Nesomyidae, Sciuridae, Thryonomyidae		0/2740 (0.0)	nd
						Eulipotyphla		Soricidae	1/159 (0.6)	Alpha
Suu-Ire *et al.* [Bibr R140][140]	2022	Africa	Ghana	T(L)	B, S	Rodentia	Muridae		0/293 (0.0)	nd
He *et al.* [Bibr R141][141]^11^	2022	Asia	China	nr	nr	Rodentia	Cricetidae		n/a ^12^	nd
						Eulipotyphla		Erinaceidae	n/a ^12^	nd
Pomorska-Mól *et al.* [Bibr R70][70]^13^	2022	Europe	Poland	V	S, T	Eulipotyphla		Erinaceidae	10/40 (25.0%)	Beta
Wasberg *et al.* [Bibr R74][74]	2022	Europe	Sweden	T(U)	T	Rodentia	Cricetidae		9/266 (3.4)	Beta
Du *et al*. [Bibr R142][142]^11^	2022	Asia	China	T(U)	T	Rodentia	Muridae		n/a ^12^	nd ^5^
Yin *et al.* [Bibr R143][143]^11^	2022	Asia	China	T(U)	F	Rodentia	Cricetidae, Muridae, Sciuridae		n/a ^12^	nd
He *et al.* [Bibr R144][144]^11^	2022	Asia	China	T(K)	F, T	Rodentia	Sciuridae, Spalacidae		n/a ^12^	nd
Zhao, Dang, Wang *et al.* [Bibr R145][145]^11^	2022	Asia	China	T(U)	T	Rodentia	Cricetidae, Muridae		n/a ^12^	nd
Wang *et al.* [Bibr R77][77]	2022	Asia	China	MF	B, S, T	Rodentia	Spalacidae		30/195 (15.4)	Alpha, Beta
						Eulipotyphla		Erinaceidae	0/20 (0.0)	nd
Chen *et al.* [Bibr R85][85]^11^	2023	Asia	China	T(U)	T	Rodentia	Cricetidae, Muridae, Sciuridae, Platacanthomyidae		n/a ^12^	Alpha, Beta
						Eulipotyphla		Soricidae	n/a ^12^	Alpha
Cui *et al.* [Bibr R146][146]^11^	2023	Asia	China	nr	nr	Rodentia	Cricetidae, Muridae, Sciuridae, Spalacidae, Talpidae, Zapodidae		n/a ^12^	Alpha, Beta
						Eulipotyphla		Erinaceidae, Soricidae	n/a ^12^	nd
Xu *et al.* [Bibr R78][78]	2023	Asia	China	T(U)	T	Rodentia	Sciuridae		1/54 (1.9)	Beta
Xu *et al.* [Bibr R147][147]	2023	Asia	China	T(L)	T	Rodentia	Cricetidae, Muridae, Sciuridae		78/439 (17.8)	Alpha, Beta
						Eulipotyphla		Soricidae	7/52 (13.5)	Alpha
Yi *et al.* [Bibr R148][148]^11^	2023	Asia	China	T(L)	B, T	Rodentia	Muridae		n/a ^12^	nd
Fisher *et al.* [Bibr R149][149]^11^	2023	Europe	UK	T(U), V	F, T	Rodentia	Muridae		n/a ^12^	n/a ^10^
Kane *et al.* [Bibr R150][150]^11^	2023	Asia	China	T(L)	T	Rodentia	Muridae		n/a ^12^	nd
Earnest *et al.* [Bibr R107][107]^13^	2023	North America	USA	T(L)	S	Rodentia	Cricetidae		5/614(0.8)	Beta
Li, Tang, Zhang, Li *et al.* [Bibr R151][151]^11^	2023	Asia	China	NR	S	Rodentia	Muridae		n/a ^12^	Beta
De Sabato *et al.* [Bibr R69][69]^13^	2023	Europe	Italy	V	F	Eulipotyphla		Erinaceidae	45/102 (44.1)^8^	Beta
Olarte-Castillo *et al.* [Bibr R152][152]	2023	North America	USA	V	F, T	Rodentia	Sciuridae		0/36 (0.0)	nd
Italiya *et al.* [Bibr R153][153]	2023	Africa	Senegal	T(L)	F	Eulipotyphla		Erinaceidae	0/20 (0.0)	nd
Arteaga *et al.* [Bibr R154][154]	2023	South America	Argentina	T(U)	F, S, T	Rodentia	Muridae		Not reported	Alpha
Apaa *et al.* [Bibr R155][155]	2023	Europe	UK	T(L), V	F, T	Rodentia	Cricetidae, Muridae		0/167 (0.0)	nd
						Eulipotyphla		Erinaceidae, Soricidae	0/5 (0.0)	nd
Wang *et al.* [Bibr R156][156]^11^	2023	North America	USA	T(L)	T	Rodentia	Muridae		1/6 (16.7)	Beta
Di Bartolo *et al.* [Bibr R157][157]	2024	Europe	Italy	T(U)	T	Rodentia	Muridae		0/128 (0.0)	nd
Wernike *et al.* [Bibr R158][158]	2024	Europe	Germany	T(U)	T, O	Rodentia	Muridae		5/130 (3.8)	Alpha
Liu *et al.* [Bibr R159][159]	2024	Asia	China	T(L)	T	Rodents	Muridae		2/74 (2.7)	Beta
Wang, Yang, Ren, Hu, Zhao *et al.* [Bibr R160][160]^11^	2024	Africa	Uganda	NR	T	Rodentia	Gliridae, Muridae, Nesomyidae, Spalacidae		n/a ^12^	nd
Domanska-Blicharz *et al.* [Bibr R71][71]^13^	2024	Europe	Poland	V	S	Eulipotyphla		Erinaceidae	5/34 (14.7)	Beta
Moreno *et al*. [Bibr R161][161]	2024	Europe	Italy	T(L)	B, F	Rodentia	Sciuridae		9/70 (12.9)	Beta
Lukina-Gronskaya, Chudinov, Korneenko *et al.* [Bibr R72][72]^11^	2024	Europe	Russia	T(L)	S	Eulipotyphla		Erinaceidae	4/21 (19.0)	Beta

1E, environmental, typically for faeces found in the environment; MF, markets and/or farms; T, trapping, either live (L), kill (K) or unspecified (U); V, volunteer submissions, such as wildlife rehabilitators and/or hunters/trappers; nr, not reported.

2Sample type used for pan-CoV screening; B, blood and/or serum; F, faeces; T, tissue; S, swabs; O, other, such as urine or saliva; nr, not reported.

3Species sampled, when available, are summarized in Table S1.

4n/a, not applicable; nd, CoVs not detected.

5Reported <10 reads of short CoV-like sequences.

6DRC, Democratic Republic of the Congo; ROC, Republic of the Congo.

7Overall CoV-positive prevalence only reported for rodents and eulipotyphlans combined; CoV genus not reported in article and retrieved manually from GenBank.

8Screening was done with an EriCoV-specific PCR.

9Used metagenomics to identify CoVs and then tested the original sample pool via PCR to investigate prevalence.

10Details of CoV-related reads not provided; authors unable to perform genome assembly.

11Metagenomic approach for pan-CoV screening; no pan-CoV PCR conducted.

12Pooled metagenomic approach; CoV+prevalence not reported at level of individual animal.

13Included statistical methods to investigate association between CoV infection and variables such as animal demographics or environmental factors.

### Small mammal coronavirus study map

To illustrate the global distribution of existing research on CoVs in wild small mammals, we created a map of the studies identified in our literature search by continent and country in ArcGIS Pro (v3.1; Esri Canada, Toronto, Ontario, Canada).

### Phylogenetic analysis

To aid in the retrieval of the numerous rodent-associated CoV sequences available, we again utilized the ZOVER database [46]. Although there are >1,600 entries of *Coronaviridae* sequences from rodent hosts, the majority of these are small partial gene sequences, typically from the well-conserved RNA-dependent RNA polymerase (RdRp) region. To ensure comparability and high-quality phylogenetic analysis, we only retrieved full-length CoV genomes and then filtered the results to exclude SARS-CoV-2 sequences and sequences recovered from virus isolation cultures. Eulipotyphlan CoV whole genomes were retrieved manually via GenBank accession numbers provided in the literature sources. In total, we compiled 90 CoV whole genomes derived from wild terrestrial small mammals, including 26 genomes from eulipotyphlan hosts and 64 genomes from rodent hosts. Multiple sequence alignment of whole genomes was performed in MAFFT using default parameters [47]; the RdRp region was manually extracted from this alignment. The spike (S) and nucleocapsid (N) regions were extracted from individual sequences according to their GenBank annotations, manually checked and subsequently aligned in MAFFT. Nucleotide substitution model tests were run in ModelTest-NG to determine the most suitable model for phylogeny construction according to Akaike information criterion, Bayesian information criterion and corrected AIC metrics [48]. Maximum likelihood phylogenies of the whole genome, RdRp, N and S regions were generated in RAxML-NG using the GTR+I+G model [49]. Phylogenies and tanglegrams were visualized in R v4.4.2 [50], using the packages treeio v1.30.0 [51], TreeTools v1.13.0 [52], phytools v2.4–4 [53], phylotools v0.2.2 [54], dplyr v1.1.4, phangorn v2.12.1 [55], ape v5.8–1 [56], ggtree v3.14.0 [57], ggimage v0.3.3 and ggplot2 v3.5.1.

## Results and discussion

### CoV discovery and diversity in small mammals

**Fig. 1.**
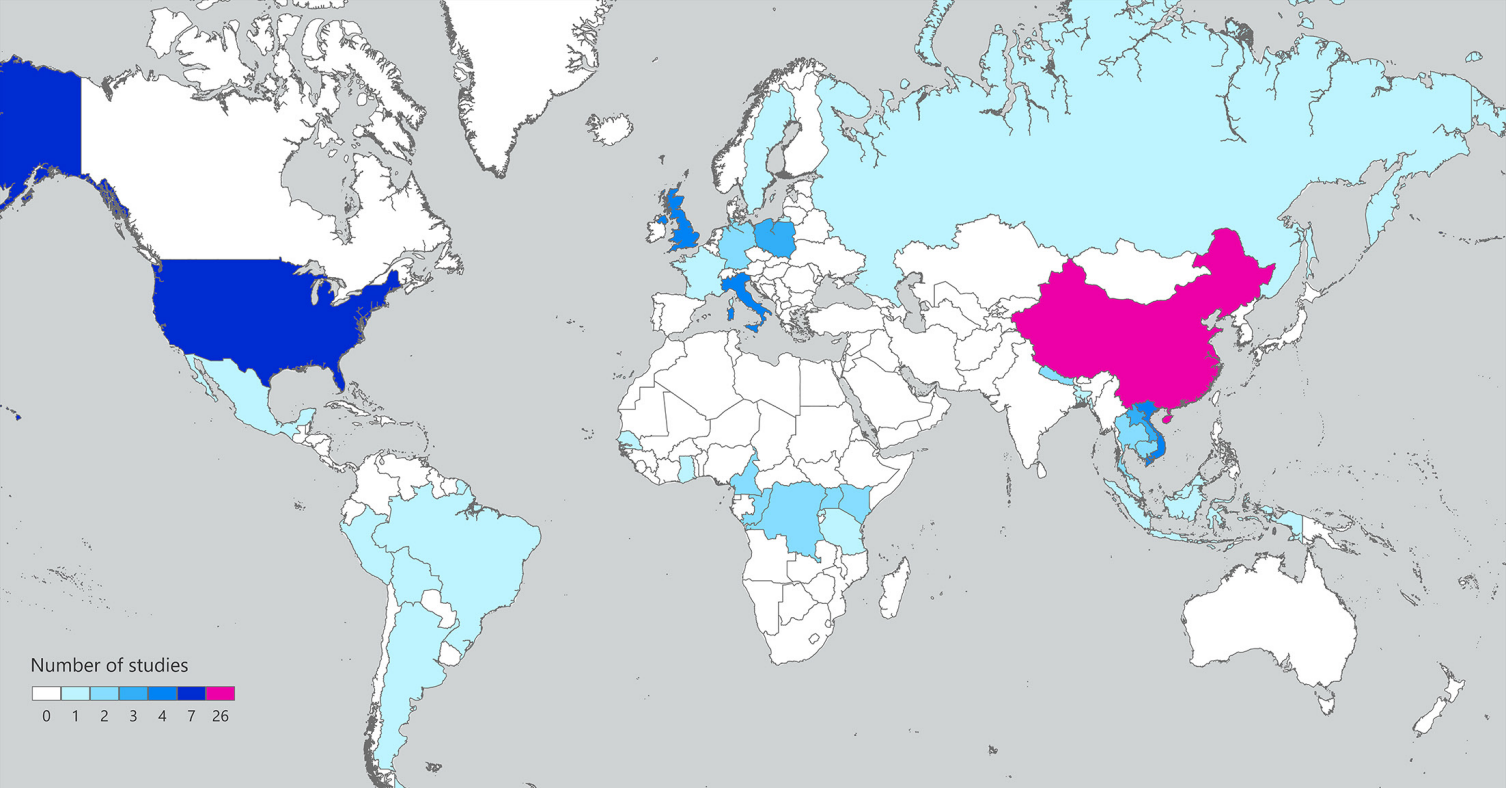
World map of wild small mammal coronavirus surveillance studies. Countries are shaded by the number of studies that have included them in their sampling; multiple studies included samples from more than one country of origin. Note that islands and regions of countries with non-contiguous borders are shaded in the same manner as their primary landmass, but this may not reflect specific areas of small mammal sampling.

From our literature search, we found 39 studies (39/63; 61.9%) that reported CoVs in wild terrestrial small mammals (Table 1). The scope of this research field has markedly progressed from what was known even a decade ago. Both the number of studies and the geographical areas sampled have increased over time. A total of 31 studies were published between 2011 and 2021, compared to 32 studies from 2022 onwards (Table 1). Most studies focused on countries within Asia (*n*=32) and Europe (*n*=15) (Fig. 1 and Table 1). Also included are eight studies from Africa, eight studies from North America and three studies from South America; no studies from Australia or Oceania were identified (Fig. 1 and Table 1).

Novel CoVs continue to be identified in wild terrestrial small mammal species, and intriguing trends are now beginning to emerge in this field. For example, CoV infection prevalence at the individual animal level can vary widely, being as high as >50% in some studies and as low as <1% in others (Table 1). Notably, most studies reporting higher CoV infection prevalence (≥25%) via PCR testing included hedgehogs only; sample sizes in these studies were typically smaller than studies that included other eulipotyphlans and rodents (Table 1). Such disparate CoV prevalences may reflect true differences across geographical regions, the families of small mammals sampled, the environments that animals are sourced from or temporal patterns in CoV infection. Alternatively, this phenomenon may be an artefact of differing sample types. While tissue and faecal samples are the most commonly used for CoV surveillance in wild terrestrial small mammals, sample type and method of sample collection are highly inconsistent across the literature (Table 1). Additionally, CoVs are known to have multiple tropisms and, therefore, limiting samples to only one type may exclude identification of some CoVs. Generally, CoVs have tropisms for epithelial tissues and are associated with enteric and respiratory infections; however, several are well-documented to cause problematic sequelae of hepatic, vascular, neurological and immunological consequences [24]. Therefore, studies that only collect and test faeces, for example, may fail to detect CoVs with a predisposition for other tissues such as respiratory or hepatic.

Species of Muridae such as the house mouse (*M. musculus*), Norway rat (*R. norvegicus*) and black rat (*Rattus rattus*) are widespread, cosmopolitan rodents that are the primary hosts for viruses within the species *Betacoronavirus muris* (previously *Murine coronavirus*), which was first described in 1949 [24,58]. *B. muris* is one of the most thoroughly studied CoVs and has been used in numerous experimental studies to study recombination of RNA viruses [59,61]. Prior to 2014 [43], few studies were published investigating CoVs harboured by wild terrestrial small mammals. A 2008 study investigated the prevalence of sialodacryoadenitis virus – a strain of *B. muris* – in wild urban rats from Baltimore, MD, USA [62]. However, this project was focused on serology only, and no testing for viral RNA or sequencing was completed. In 2011, a small survey aimed to characterize the total viral diversity in faeces of rodents from the USA and reported exceedingly few sequencing reads classified as *Coronaviridae* (Table 1) [63]; no further information on CoV sequencing reads was provided. The first intentional and direct characterizations of CoVs infecting wild terrestrial small mammals involved studies by Corman *et al*. in 2014, Wang *et al*. in 2015 and Lau *et al*. in 2015, which investigated CoVs present in small mammals from western Europe and southeast Asia [43,64, 65].

In 2014, Corman *et al*. tested the faeces of 248 European hedgehogs (*Erinaceus europaeus*) from Germany and discovered four viruses that were subsequently designated *Betacoronavirus erinacei* (previously *Hedgehog coronavirus 1*) and colloquially termed EriCoVs [43]. These viruses clustered phylogenetically with beta-CoVs recovered from bats. Specifically, EriCoVs shared high genome similarity with MERS-CoV and other MERS-related CoVs – an intriguing discovery especially given the high prevalence of EriCoVs (146/248; 58.9%) in the hedgehogs sampled [43]. Other EriCoVs possessing >90% nucleotide identity with *B. erinacei* have since been identified in European hedgehogs from the UK, Italy, Poland and Russia [66,72]. Studies of Amur hedgehogs (*Erinaceus amurensis*) from Hong Kong and China have identified another MERS-related CoV, currently named *Erinaceus* hedgehog coronavirus HKU31 [44,45].

Shortly following the work by Corman *et al*., Wang *et al*. published findings of novel CoVs in wild rodent species from both urban and rural locations in China [64]. Based on genome percent-nucleotide identity, the beta-CoVs that were detected were categorized into: Longquan Aa mouse CoV (LAMV), found in the striped field mouse (*Apodemus agrarius*) and determined to be a strain of the species *Betacoronavirus gravedinis* (previously *Betacoronavirus 1*), and Longquan Rl rat CoV (LRLV), found in the lesser ricefield rat (*Rattus losea*) and determined to be a strain of *B. muris* [64]. In contrast, the highly interrelated alpha-CoV genomes found in Norway rats (*R. norvegicus*) from this study were sufficiently distinct to be classified as a novel species, designated *Alphacoronavirus ratti* [previously *Lucheng Rn rat coronavirus* (LRNV)] [64]. Upon constructing phylogenies with these viruses, LAMV and LRLV consistently clustered with beta-CoVs, but the placement of LRNV differed substantially depending on which gene was used for tree construction. For example, LRNV clustered with alpha-CoVs when phylogenies were constructed using the 3 CL, Hel, RdRp and S regions but formed a distinct beta-CoV lineage when examined using the N gene [64]. Furthermore, LRNV clustered with different alpha-CoVs for each of the 3 CL, Hel, RdRp and S, including alpha-CoVs from bats, mink and domestic cats [64]. The authors performed further analysis to identify recombination events using RDP4 [73]. Notably, multiple statistically significant recombination events were detected involving LRLV strain Longquan-708 (GenBank accession no. KF294372.1) and two rodent CoV parent genomes (murine CoV MHV-1, FJ647223.1; LRLV Longquan-370, KF294371.1) [64]. The work of Wang *et al*. included the first report of an alpha-CoV in a *Rattus* sp. but was also the first of many instances to provide strong evidence for recombination in CoVs of wild terrestrial small mammals [64].

Also in 2015, Lau *et al*. conducted pan-CoV PCR screening of 664 wild rodents and shrews, with the majority of samples collected from Norway rats (*R. norvegicus*; *n*=390; 53.8% of samples); only five shrews were tested [65]. Three Norway rats, all from the same location, were PCR-positive; three nearly identical genomes were generated and were identified as a novel divergent beta-CoV species named *Betacoronavirus ratti* (previously *China Rattus coronavirus HKU24*). The authors of this study performed a molecular clock analysis that suggested *B. ratti* emerged prior to *B. gravedinis* viruses [65]. As such, Lau *et al*. hypothesized that *B. ratti* could represent a common ancestor for *B. gravedinis* viruses [65].

There is a wide diversity of CoVs that infect wild rodents and eulipotyphlans. Originally, it was thought that rodents alone were involved in the ecology of just a handful of beta-CoVs, but it is now evident that numerous CoVs of several genera infect both rodent and eulipotyphlan small mammal hosts (Table 1). Our literature search identified 17 studies (17/63; 27.0%) that reported alpha-CoVs, 33 studies (33/63; 52.4%) that reported beta-CoVs and one study (1/63; 1.6%) that reported a delta-CoV; eleven of these studies (11/63; 17.5%) reported CoVs of multiple genera (Table 1). These viruses were split between hosts of both rodent and eulipotyphlan taxonomy. Small mammal genera and species tested, when such information was available, are summarized in Table S1, available in the online Supplementary Material.

**Fig. 2.**
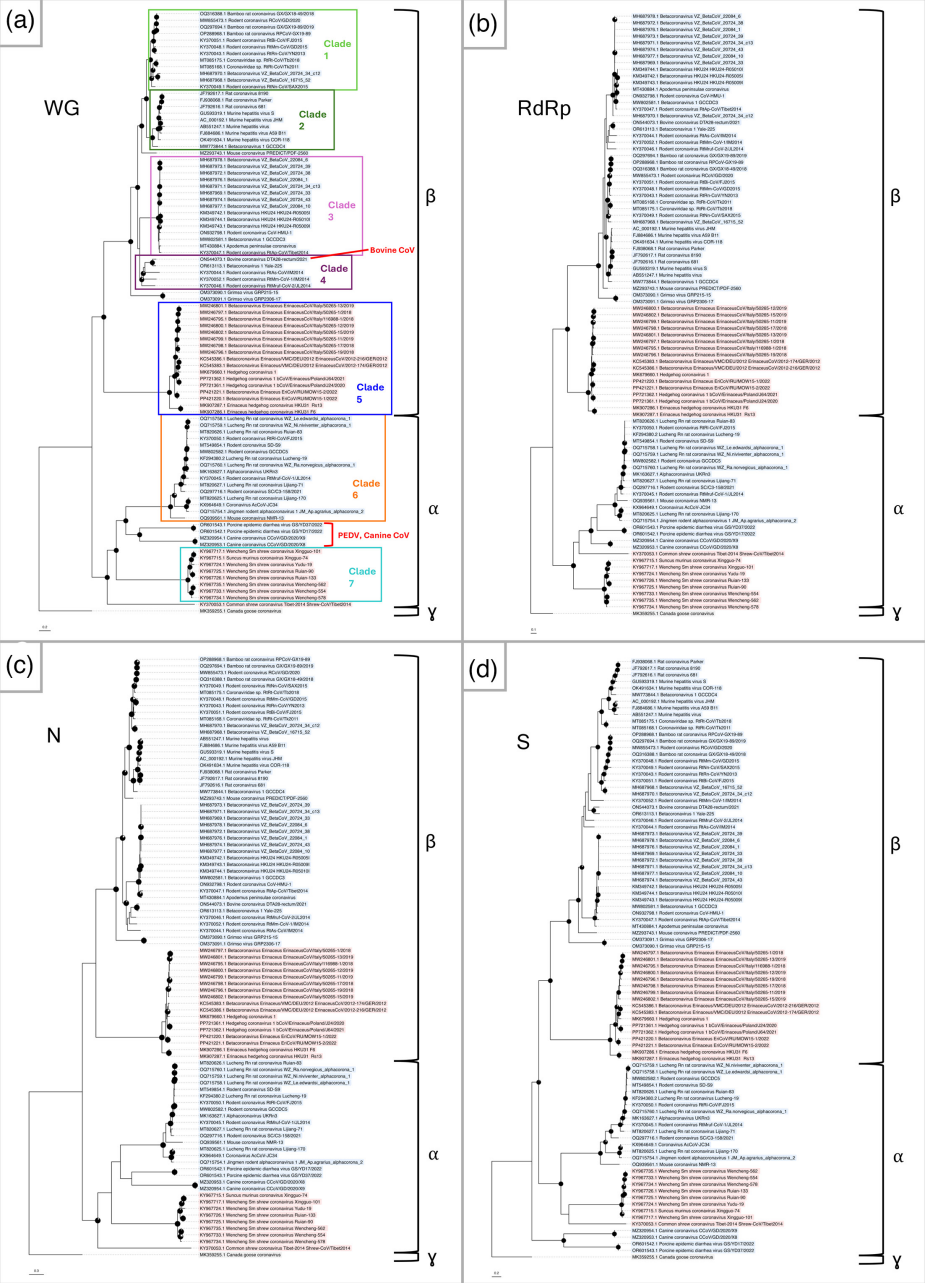
Phylogenetic analyses of (**a**) complete genome sequences, (**b**) RNA-dependent RNA polymerase (RdRp), (**c**) nucleocapsid (N) and (**d**) spike (S) regions from coronaviruses recovered from wild rodent and eulipotyphlan small mammals. Sequences recovered from rodents and eulipotyphlans are highlighted in blue and red, respectively; a gammacoronavirus outgroup (Canada goose coronavirus) is highlighted in grey. Well-supported clades in the whole-genome phylogeny, as described in the text, are indicated with different coloured boxes in panel A. Maximum likelihood trees were estimated in RAxML-NG using a GTR+I+G substitution model. Support was calculated with bootstrap analysis (500 replicates) and indicated at nodes with pie charts; support values <75 are not shown. GenBank accession numbers are provided for each sequence. Scale bar indicates phylogenetic distance (nucleotide substitutions per site).

**Fig. 3.**
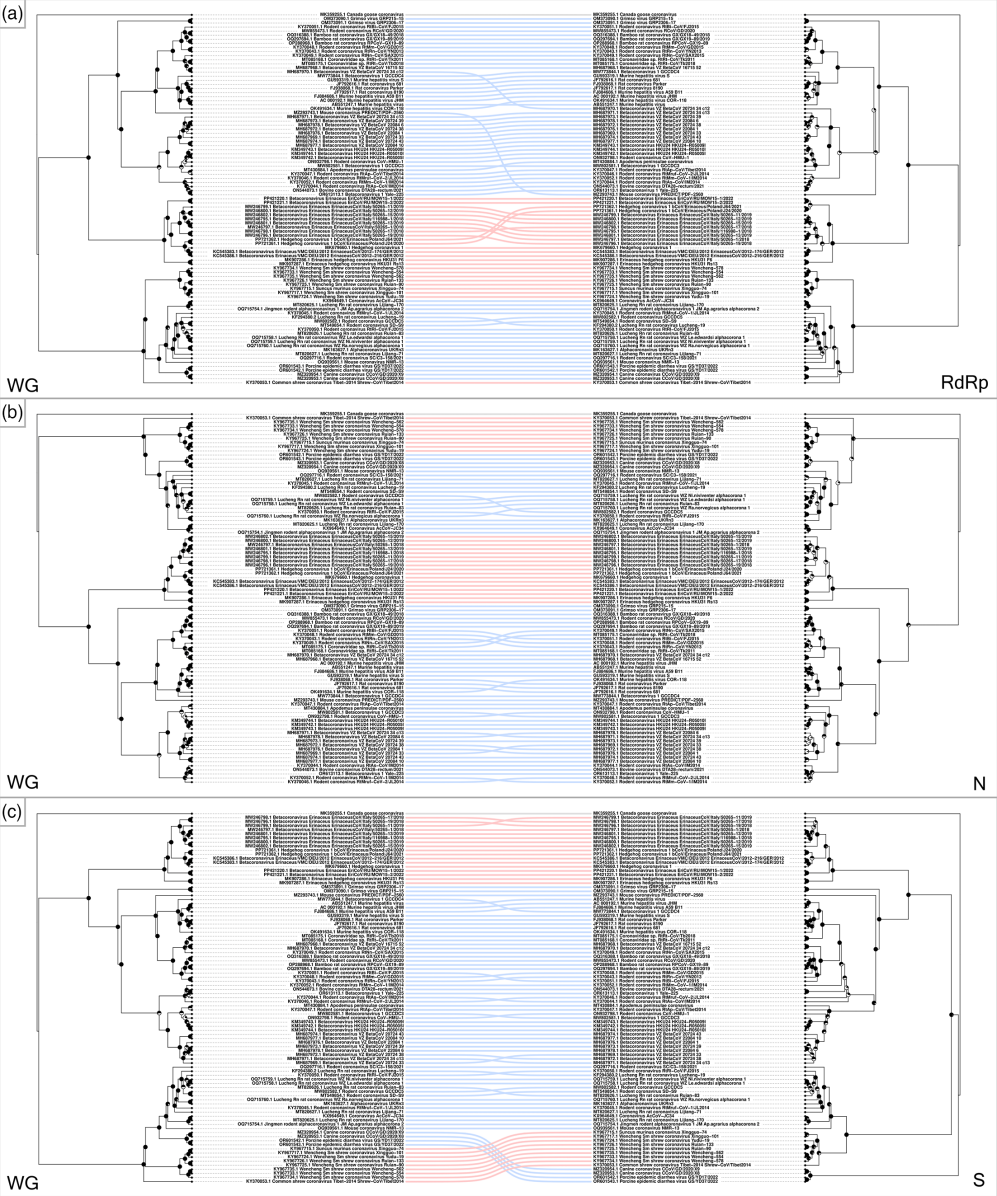
Tanglegrams of maximum likelihood phylogenies of coronavirus whole genomes (left) and (**a**) RNA-dependent RNA polymerase (RdRp), (**b**) nucleocapsid (N) and (**c**) spike (S) regions (right) obtained from wild rodent and eulipotyphlan small mammals. Linkages between phylogeny tips for rodents (blue) and eulipotyphlans (red) show differences in topologies; a gammacoronavirus outgroup (Canada goose coronavirus) is linked in grey. Phylogenies were estimated in RAxML-NG using a GTR+I+G substitution model; support values calculated with bootstrap analysis (500 replicates) are depicted with pie charts at nodes.

**Fig. 4.**
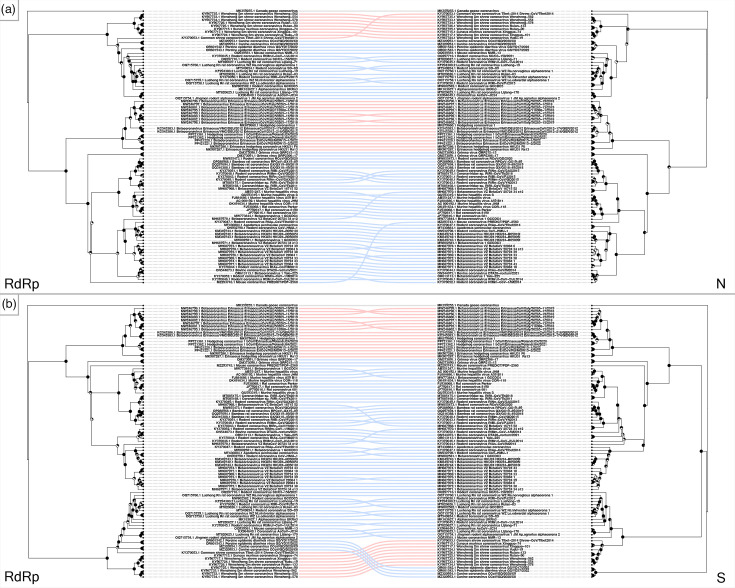
Tanglegrams of maximum likelihood phylogenies of coronavirus RNA-dependent RNA polymerase (RdRp) regions (left) and (**a**) nucleocapsid (N) and (**b**) spike (S) regions (right) obtained from wild rodent and eulipotyphlan small mammals. Linkages between phylogeny tips for rodents (blue) and eulipotyphlans (red) show differences in topologies; a gammacoronavirus outgroup (Canada goose coronavirus) is linked in grey. Phylogenies were estimated in RAxML-NG using a GTR+I+G substitution model; support values calculated with bootstrap analysis (500 replicates) are depicted with pie charts at nodes.

Similar to what is described in literature, our phylogenetic analysis conducted on available CoV whole genomes recovered from wild terrestrial small mammals presents a diversity of both alpha- and beta-CoVs (Fig. 2a). Rodent beta-CoVs are structured into four major well-supported clades (denoted clades 1–4 for the purposes of discussion) and a more basal lineage, including Grimso viruses recovered from bank voles (*Clethrionomys glareolus*) in Sweden (Fig. 2a) [74]. Rodent alpha-CoVs form a single well-supported clade (Fig. 2a, clade 6). Eulipotyphlan beta-CoVs form a single clade, including viruses obtained from *Erinaceus* hedgehogs (EriCoVs) as well as *Erinaceus* hedgehog coronavirus HKU31 (Fig. 2a, clade 5). Eulipotyphlan alpha-CoVs (Fig. 2a, clade 7) are limited to those from shrew species (family Soricidae); however, all but one of the whole genomes available came from a single study [75], and the other [76] appears to represent a basal lineage of this clade. A small group of alpha-CoVs recovered from rodents composed of CoVs not typically associated with rodent hosts (Fig. 2), including canine CoV [77] and porcine epidemic diarrhoea virus (PEDV), should be noted (unpublished, GenBank accession nos. OR601542.1 and OR601543.1, Fig. 2a). Additionally, a strain of bovine CoV recovered from a Daurian ground squirrel (*Spermophilus dauricus*) [78] is present in the rodent beta-CoV branch of the phylogeny (Fig. 2a). These may represent examples of recent cross-species transmission events from larger mammals into rodent hosts. The shortened phylogenetic distance of the bovine CoV sequence to other rodent beta-CoVs versus that of the aforementioned canine and porcine alpha-CoVs to known small mammal alpha-CoVs is also noteworthy (Fig. 2). The overall topology seen in the whole genome phylogeny remains largely unchanged in phylogenies of the RdRp, N and S regions alone (Fig. 2b–d); this is reflected in direct comparisons using tanglegrams (Fig. 3). The majority of incongruences, albeit minor, are seen in comparisons of the whole genome to the spike and nucleocapsid regions (Fig. 3b, c). This is unsurprising given the fact that multiple studies have suggested these regions to be the most variable in CoVs of terrestrial small mammals, perhaps being more prone to recombination [64,76, 79]. Changes in topology are more significant when comparing the RdRp region to the N and S genes, where sequence clustering moves between major clades (Fig. 4). This may reflect greater genome plasticity in the structural protein-coding regions near the 3′ end of CoV genomes, relative to ORF1ab, potentially driven by higher mutation rates and more frequent recombination in these regions [80,83].

Previous research has suggested that alpha-CoVs and beta-CoVs of rodents and eulipotyphlans have low host species specificity and are prone to frequent host switching events [79,84, 85]. This contrasts with observations of CoVs in bat hosts, where CoVs are almost exclusively reported to exhibit high host specificity to individual bat species [84,86,90]. The frequent and efficient interspecies transmission of CoVs infecting rodent and eulipotyphlan small mammal hosts likely contributes to the evolutionary trajectory and resultant high diversity of these viruses. The seemingly promiscuous nature of terrestrial small mammal CoVs can contribute to CoV evolution through two main mechanisms: (1) host–virus interactions in novel hosts resulting in host adaptation and (2) recombination [91,95]. Recombination occurs during genome replication via spontaneous template switching between two parent genomes that co-infect the same cell [96]. Resultant recombinants represent mosaic viruses that can bear consequences for virus host range, pathogenicity, transmissibility and immune evasion [33,91, 96]. Co-infection with multiple CoV strains or species has been previously observed in laboratory mice and wild bats, suggesting rodent and chiropteran hosts could act as mixing vessels for CoV genome recombination in a similar fashion to swine hosts acting as reassortment foci for influenza viruses [60,97, 98]. At least 12 studies identified in our literature search performed recombination analyses on the viruses they discovered; over half (8/12; 66.7%) reported strong evidence of recombination events [64,65, 68, 71, 74, 75, 77,79, 84, 99, 100]. It is important to recognize that many terrestrial small mammal taxa remain sparsely sampled, even in CoV sampling hotspots such as eastern Asia. Thus, some caution must be exhibited when inferring recombination events across large geographical scales since such events may represent undersampling of the full phylogenetic gradient of these viruses.

Co-infection with multiple CoVs in terrestrial small mammals has been documented in a Vietnam study by Huong *et al*. where wild rats sampled from live animal markets and restaurants were shown to be co-infected with different beta-CoVs [98]. In the same study, rodent faeces taken from commercial rodent farms were found to contain unexpected CoVs, including avian gamma-CoVs and bat alpha-CoVs [98]. The authors noted that the rodent farm where bat and avian CoVs were detected in rodent faeces may have had bats and birds roosting within the premises [98]. This study provided clear examples of scenarios where environmental mixing of, exposure to, and co-infection with different CoVs in rodent and eulipotyphlan small mammal hosts could occur. Other studies have demonstrated the presence of unexpected CoVs in rodent hosts, such as bovine CoVs, canine CoVs and deltacoronaviruses [77,78, 100]. Such scenarios demonstrate opportunities for virus co-occurrence; possible recombination of CoVs in wild terrestrial small mammals may be more common than previously recognized.

Total reported CoV-positive animals differed notably between studies that employed targeted PCR-based screening approaches to those that used a metagenomics approach. Only 10 of the 22 (45.4%) studies identified in our search that used metagenomics-based pan-CoV screening reported CoV-positive animals, compared to 35/41 (85.4%) for PCR-based studies. Publication bias may be a contributing factor since identifying CoVs was often not the primary research objective in studies with metagenomic-based screening. Regardless, the use of sample pooling for efficiency and cost-effectiveness of CoV screening subsumes individual-level CoV infection status. Unless samples are followed up on an individual basis, sample pooling precludes calculating CoV prevalence in small mammal communities being sampled, which is a valuable metric for identifying regional differences in prevalence. This is particularly the case for studies that, for example, have tested the same species of small mammals across different environments, or have tested the same small mammal communities longitudinally. However, metagenomic-based approaches can allow for immediate recovery of whole CoV genomes, obviating the need for additional sequencing steps after PCR screening.

### Spillover of small mammal CoVs

The analysis of non-structural ORFs and accumulated gene losses across genomes of alpha- and beta-CoVs, particularly merbecoviruses and sarbecoviruses, has provided evidence that the majority of human CoV pathogens diverged from strains found in bats [34]. However, more recent work has focused on the role of small mammal CoVs as emerging human pathogens. Notable embecoviruses identified as common and widespread seasonal respiratory pathogens, such as human CoV OC43 and *Betacoronavirus hongkongense* (previously *Human coronavirus HKU1*), likely have rodent origins [34,65, 101,103]. Specifically, multigene phylodynamics and Markov host-jump analyses have provided robust support for a rodent origin of *B. hongkongense* [102,103]. The original hypothesis of human CoV OC43 emergence was a spillover event from a bovine host [104,105]. However, newer analysis suggests a more complex origin of OC43 involving rodent, rabbit and ungulate hosts as well [103], and the recent identification of OC43-related embecoviruses from wild *Peromyscus* mice (family Cricetidae) in the USA supports this idea [106,107]. The discovery of species *B. ratti* suggested a longstanding co-evolutionary relationship of endemic rodent CoVs with their hosts [65]; this hypothesis was supported by virus–host cophylogenetic analyses by Tsoleridis *et al*. and Wu *et al*. [76,79].

Furthermore, many embecoviruses have been identified as possessing polybasic cleavage sites within the adjoining region between the spike protein subunits S1 and S2 [102]. An analysis using multiple prediction models of polybasic cleavage sites reported that 78% of evaluated rodent CoV spike sequences contained predicted furin cleavage sites in the S1/S2 region, compared to just 6% in bat sequences [102]. A polybasic cleavage site in the S1/S2 region is an important virulence factor of SARS-CoV-2, allowing enhanced cell entry, cell-cell fusion and immune evasion [108,110]. This concept has been previously demonstrated with avian influenza viruses, where the presence of multiple polybasic cleavage sites significantly contributes to the virulence of highly pathogenic avian influenza strains [111,112]. This presents important molecular evidence that may bear significant implications for the permissibility of rodent CoVs to infect and cause severe disease in humans and other species.

In February 2025, Park *et al*. described a novel coronavirus causing disease in a human from South Korea [113]. The case, from December 2022, details an infant admitted to the hospital with flu-like respiratory illness and subsequent liver dysfunction. PCR testing of nasal swabs and serum revealed infection with human parainfluenza virus type 1 and rhinovirus; subsequent viral metagenomic sequencing revealed the presence of a concomitant CoV designated HCoV KUMC22-3# [113]. This CoV was most similar (95.0% nucleotide identity at the genome level) to AcCoV-JC34, an alpha-CoV recovered from rodents in China [114]. Subsequent testing of rodent faecal samples from South Korea revealed similar alpha-CoVs with genome nucleotide identity of 93.0–96.8% to HCoV KUMC22-3# [113]. This was reflected in similar protein coding, including two rodent CoVs possessing envelope (E) proteins with 100% amino acid identity to HCoV KUMC22-3# [113]. This is relevant given the importance of the E protein in the virulence of other CoVs [115,117]. The case report and subsequent investigation described by Park *et al*. [113] is a current example of the spillover potential of CoVs from wild terrestrial small mammals, underscoring the importance of further surveillance in these hosts.

### Ecology, surveillance and One Health

Although work has been completed to characterize known rodent and eulipotyphlan CoVs on a structural, genomic and evolutionary basis, there is much to be examined regarding the complex and arguably cryptic ecology of these viruses. Coronaviruses carried by small mammals represent a potential zoonotic risk, especially given the peridomestic nature of these animals and their frequenting of habitats used by other animals such as companion animals and livestock. This is particularly relevant for multiple facets of the transmission dynamics of endemic small mammal pathogens, including viruses. For example, rodents are well known to forage for seeds and undigested plant material in the faeces of other, larger mammals as well as conspecifics [118,119]. This provides a direct means of faecal–oral transmission of CoVs to small mammals from other wildlife or domestic animals. In the case of livestock agriculture, small mammals may be exposed to the endemic CoVs of animals such as cattle, swine, horses and geese. A possible example of this process has been suggested for swine acute diarrhoea syndrome coronavirus, as there is some suggestion of rodents playing a role in the emergence or transmission of this virus on swine farms [120]. Given what is known regarding the frequent recombination of CoVs, there should be consideration given to the possibility of novel recombinants being generated in small mammal hosts in livestock agriculture environments. Perhaps such a situation contributed to the evolution of a highly similar group of beta-CoVs, as seen in multiple analyses of *B. gravedinis*-like virus sequences, including those from bovine, rodent, porcine and human hosts [103,105]. Our phylogenetic analysis also supports this theory, with a bovine CoV recovered from a rodent being closely clustered to other rodent beta-CoVs, both at the whole genome and RdRp level (Fig. 2ab).

Considering the ubiquity of wild terrestrial small mammals and their predisposition for peridomestic habitats, it is important to consider the environmental contexts where CoV discovery studies are being conducted and how samples are collected. While many studies have conducted trapping or collected samples from pre-existing surveillance or hunting programmes, some research has focused on animals involved in the human food chain. In certain countries, farming of animals taken from the wild, such as the Chinese bamboo rat (*Rhizomys sinensis*), has opened an avenue for amplification of potential pathogens carried by these animals as well as an intensified human-animal interface [77]. At live animal markets, where an abundance of other wildlife and domestic species may be present, opportunities can arise for the exchange of CoVs between animals [39,121]. Several research teams have sampled rodents from wildlife farms, meat markets, restaurants and animal traders [65,98, 122,126]. Based on these results, researchers have suggested that CoVs in wild terrestrial small mammals are just as prevalent in food chain settings as they are in naturalized areas; notably, Huong *et al*. showed that the prevalence of CoVs in wildlife overall may increase along the human food chain gradient [98].

Unfortunately, many authors investigating CoV diversity in wild small mammals include little detail regarding their study environments and sample collection methods or neglect to include specifics of sampling procedures altogether. In future studies, including descriptions of where samples originate and how they were collected will allow for proper contextualization of CoV surveillance and characterization of results. Examining the interfaces where wild terrestrial small mammals exist and the ecological processes they are subject to will allow researchers to begin unravelling the natural transmission cycles of wildlife CoVs. Where possible, researchers should also consider the analytical methods used for assessing the association of different factors with CoV infection in wild small mammals. Indeed, only ten of the studies identified in our literature search (16.7%, Table 1) included any form of statistical measures of association between CoV infection and variables such as animal demographic factors, including age, sex or species, or environmental factors like urban or rural location type.

As previously discussed, inconsistency in the type(s) of samples used for CoV screening translates to varying sensitivity and potential failure to identify co-infections, depending on virus tropism. Consistency of sample types across host species and collection sources will increase comparability between studies. Including multiple sample types that target different tissue types will reduce false negatives in CoV screening of small mammals. Organ and tissue samples, while common – 44/63 (69.8%) of studies in our literature search reported using tissue and/or blood samples – are not necessarily the most ideal approach for CoV surveillance. Some studies (*n*=17; 27.0%) used only swabs and/or faeces and 16/17 were able to identify CoVs (Table 1). Therefore, using less-invasive sample types such as swabs or faeces does not appear to markedly decrease sensitivity for detecting CoVs. Proper storage of samples and use of high-quality transfer media aid in preserving sample quality and diagnostic sensitivity. Furthermore, screening methodology may play an important role in surveillance. As discussed previously, utilizing a robust and validated PCR-based protocol for screening small mammal samples will provide a high-sensitivity approach when investigating CoVs. Finally, as whole-genome sequencing techniques become more cost-accessible, research teams should opt for this practice when possible. While many studies do provide at least partial sequence data, typically for a fragment of the well-conserved RdRp region of ORF1b, CoVs are highly variable within other portions of the genome. Building on the existing base of CoV whole genomes recovered from wild terrestrial small mammals will allow for an increased understanding of the diversity and relatedness among these viruses, better detection of recombination events and enhanced specificity of surveillance programmes overall. Furthermore, the base of knowledge for wild rodent and eulipotyphlan CoVs will be made more comparable to that of bats – only ~90 whole genomes of rodent and eulipotyphlan CoVs currently exist in NCBI GenBank compared to >500 genomes for bat CoVs [46].

The results of disease surveillance studies targeting wild rodent and eulipotyphlan small mammal hosts are also relevant for the health of humans and domestic animals, as discussed here. Hence, studies in this field should be undertaken using a One Health approach. A One Health approach is multi-disciplinary and includes cross-sectoral considerations in implementation [127]. Zoonotic diseases and their transmission are complex and inherently involve the health of humans, wild animals, domestic animals and the environment. This is particularly the case in urban ecosystems that are heavily modulated by anthropogenic activities and contain abundant human-animal interfaces. When feasible, novel or existing CoV surveillance programmes should strive to include highly qualified personnel in the areas of human public health and clinical surveillance, domestic animal health, livestock health, wildlife management and ecology, environmental science and ecosystem health, and virology and molecular diagnostics [127]. This list is non-exhaustive but provides the basics of an effective One Health structure for zoonotic virus surveillance and characterization.

## Conclusion

Research, surveillance and management of animal CoVs requires a One Health approach that considers the health of humans, wildlife, domestic animals and the ecosystems where they coexist. While research into the diversity of these viruses in bats is abundant, similar investigations in rodents and eulipotyphlans are comparatively limited. This is particularly so in the Americas, Australia and Oceania where, despite diverse populations of wild terrestrial small mammal species, only 13.3% of the identified studies in this review were conducted (Fig. 1 and Table 1). However, in the last decade, the number of studies of CoV diversity in rodent and eulipotyphlan small mammals has been increasing. While thorough work has been done to characterize the viruses identified, major gaps remain in understanding the ecology of these viruses in their hosts. Elucidating environmental and demographic factors involved in the reservoir ecology and sylvatic transmission of potentially zoonotic CoVs can allow steps to be taken for avoiding spillover to humans or controlling spread among animal populations [128]. Such steps include identifying target host species, environmental interfaces and sample types for further CoV surveillance. It is clear that CoVs present a zoonotic threat. If the ecology of these viruses remains poorly understood, then novel spillover events – whether to humans or other species – will continue to be a threat.

## Supplementary material

10.1099/jgv.0.002130 Uncited Table S1.

## Abbreviations

alpha-CoVs: *Alphacoronavirus*
beta-CoVs: *Betacoronavirus*
COVID-19: Coronavirus disease 2019
delta-CoVs: *Deltacoronavirus*
E: envelope
gamma-CoVs: *Gammacoronavirus*
LAMV: Longquan Aa mouse CoV
LRNV: Lucheng Rn rat coronavirus
MERS-CoV: Middle East respiratory syndrome-related coronavirus
N: nucleocapsid
RdRp: RNA-dependent RNA polymerase
S: spike
SARS-CoV-2: severe acute respiratory syndrome coronavirus 2
SARS-CoV: severe acute respiratory syndrome-related coronavirus
ZOVER: zoonotic and vector-borne viruses
